# Description of two new filtering carnivore *Drusus* species (Limnephilidae, Drusinae) from the Western Balkans

**DOI:** 10.3897/zookeys.513.9908

**Published:** 2015-07-16

**Authors:** Simon Vitecek, Mladen Kučinić, János Oláh, Ana Previšić, Miklós Bálint, Lujza Keresztes, Johann Waringer, Steffen U. Pauls, Wolfram Graf

**Affiliations:** 1 Department of Limnology and Bio-Oceanography, University of Vienna, Althanstrasse 14, A-1090 Vienna, Austria; 2 Department of Biology, Faculty of Science, University of Zagreb, Rooseveltov trg 6, HR-10000 Zagreb, Croatia; 3 Tarján u. 28, H-4032 Debrecen, Hungary; 4 Senckenberg Biodiversity and Climate Research Centre (BiK-F), Senckenberganlage 25, D-60325 Frankfurt a.M., Germany; 5 Hungarian Department of Biology and Ecology, Babeş-Bolyai University, Clinicilor 5–7, 400006 Cluj-Napoca, Romania; 6 Institute of Hydrobiology and Aquatic Ecology Management, University of Natural Resources, Max-Emanuelstrasse 17, A-1180 Vienna, Austria

**Keywords:** Caddisfly, aquatic diversity, Mediterranean, taxonomy, conservation, Southern Europe

## Abstract

Two new species of the genus *Drusus* (Trichoptera, Limnephilidae, Drusinae) from the Western Balkans are described. Additionally, observations on the biodiversity and threats to the region’s endemic aquatic fauna are discussed. *Drusus krpachi* **sp. n.** is a micro-endemic of the Korab Mountains, Macedonia, and *Drusus malickyi* **sp. n.** is a micro-endemic of the Prokletije Mountains, Albania. Both new species are most similar to *Drusus macedonicus* but differ from the latter in the shape of segment IX, the shape of the tips of the intermediate appendages in lateral view, the shape of the inferior appendages, and the form and shape of the parameres. In addition, males of the European species of filtering carnivore Drusinae are diagnosed and illustrated, including *Cryptothrix nebulicola* McLachlan, *Drusus chrysotus* Rambur, *Drusus discolor* Rambur, *Drusus macedonicus* Schmid, *Drusus meridionalis* Kumanski, *Drusus muelleri* McLachlan, *Drusus romanicus* Murgoci and Botosaneanu, and *Drusus siveci* Malicky. These additions to the Western Balkan fauna demonstrate the significance of this region for European biodiversity and further highlight the importance of faunistic studies in Europe.

Dedicated to Hans Malicky on the occasion of his 80th birthday

## Introduction

The Western Balkans harbour high biodiversity including high numbers of endemic species. This has been attributed to historic climate conditions and the highly diverse geology of the region (e.g., Neubauer 2002, Reed et al. 2004, Chiari et al 2011) that permitted perseverance of taxa in glacial refugia (e.g., Tzedakis 2004, 2009; Médail and Diadema 2009), and the formation of diverse habitats. Thus, the Western Balkans are rich in endemic plant (e.g., Eastwood 2004, Petrova and Vladimirov 2010, Mereďa et al. 2011, Redžić 2011), vertebrate and invertebrate species (e.g., Bianco 1998, Bănărescu 2004, Kryštufek 2004, Griffiths and Frogley 2004, Bohlen et al. 2006, Guéorguiev 2007, Deltshev 2008, Pešić and Glöer 2013). The Western Balkans also have been identified as a hotspot of aquatic biodiversity, with high endemism and cryptic diversity (Bănărescu 2004, Previšić et al. 2014a). Climate change and its detrimental effects on biodiversity (e.g., Hering et al. 2009, Bálint et al. 2011) have focused attention on freshwater biota throughout Europe, including the Western Balkans (e.g., Zakšek et al. 2009, Klobučar et al. 2013, Weiss et al. 2014).

Faunal studies on Western Balkan aquatic biodiversity recovered intriguing biogeographic patterns and several new species (e.g., Petkovski et al. 2009, Pešić and Glöer 2013, Vitecek et al. 2015). Research on the caddisfly fauna of the Western Balkans further suggests several factors, such as karstification, as drivers of speciation (Previšić et al. 2009, 2014b). The limnephilid subfamily Drusinae is particularly species-rich in the Western Balkans, including a high proportion of micro-endemics *sensu* Graf et al. (2008), i.e., taxa restricted to small geographic units within an ecoregion *sensu* Illies (1978) (Malicky 2004; Graf et al. 2008; Oláh 2010, 2011; Kučinić et al. 2011a,b; Oláh and Kovács 2013; Previšić et al. 2014a, b; Vitecek et al. 2015; Ibrahimi et al. pers. comm.).

The subfamily Drusinae Banks comprises roughly 110 species in 8 genera (Malicky 2004, 2005; Oláh 2010, 2011; Oláh and Kovács 2013; Previšić et al. 2014a; Vitecek et al. 2015; Ibrahimi et al. pers. comm.). Ecologically, most species are crenobiont (Graf et al. 2008), and as larvae fall into one of three different feeding groups: filtering carnivores, omnivorous shredders and scraping grazers (Pauls et al. 2008, Graf et al. 2009). The adults of each larval feeding group are also characterized by a set of synapomorphies (Vitecek et al. in press). Filtering carnivorous Drusinae males uniquely exhibit laterally positioned gland openings at the fifth abdominal sternite and parallel wing veins in the hind wing anal field (depicted in Vitecek et al. in press). The largest genus *Drusus* is paraphyletic (Pauls et al. 2008) and comprises 86 species of all feeding types (Malicky 2004, 2005; Graf et al. 2008; Kučinić et al. 2011a; Oláh 2010, 2011; Oláh and Kovács 2013; Vitecek et al. 2015; Vitecek et al. in press; Ibrahimi et al. pers. comm.). The monotypic genus *Cryptothrix* (*Cryptothrix nebulicola* McLachlan) is also a filtering carnivore (Bohle 1987, Graf et al. 2008), and thus represents another filtering carnivorous Drusinae *sensu* Pauls et al. 2008, the systematic position of which is discussed in Vitecek et al. (in press).

Here we describe two new filtering carnivore *Drusus* species. Additionally, we provide re-descriptions of filtering carnivorous Drusinae *sensu* Pauls et al. (2008) in order to facilitate identification of known filtering carnivorous Drusinae, and identification of new species.

## Materials and methods

Adults were collected using sweep nets and by handpicking. Collected specimens were stored in 96% EthOH. Male and female genitalia were examined after being cleared in either KOH or lactic acid. Nomenclature of male genitalia of *Drusus* follows Nielsen (1957, for *Limnephilus flavicornis* Fabricius) using the simplifying terms “superior appendages” for the lateral processes of segment X (cerci *sensu* Snodgrass 1935), and “intermediate appendages” for the sclerite and the anterior process of segment X (paraproct *sensu* Snodgrass 1935). Nomenclature of larval morphological features follows Wiggins (1998) and Waringer and Graf (2011), nomenclature of primary setae and setal areas follows Wiggins (1998). Illustrations were prepared according to Thomson and Holzenthal (2010) in which pencil drawings made with a camera lucida are digitized, edited and digitally inked in Adobe Illustrator (v. 16.0.4, Adobe Systems Inc.).

Specimens are currently stored in the following collections: Collection Wolfram Graf (WG), Institute of Hydrobiology and Aquatic Ecology Management, University of Natural Resources, Max-Emanuelstrasse 17, A-1180 Vienna, Austria; Collection Ana Previšić (AP), Department of Biology, Faculty of Science, University of Zagreb, Rooseveltov trg 6, HR-10000 Zagreb, Croatia; Collection Mladen Kučinić (MK), Department of Biology, Faculty of Science, University of Zagreb, Rooseveltov trg 6, HR-10000 Zagreb, Croatia; Collection János Oláh [János Oláh Private Collection under national protection of the Hungarian Natural History Museum, Budapest, Hungary] (JO), Tarján u. 28, H-4032 Debrecen, Hungary.

Type specimens will be deposited in museum collections upon completion of the taxonomic work.

## Taxonomy

### Descriptions of the new species

#### Drusus krpachi

Taxon classificationAnimaliaTrichopteraLimnephilidae

Kučinić, Graf & Vitecek sp. n.

http://zoobank.org/74BBEB74-1232-4B4E-934B-D8BE6433DDCB

Fig. 1

##### Material examined.

**Holotype.** 1 male: Macedonia, Mavrovo National Park, Korab Mountains, česma Elem; N41.857, E 20.625; leg. Kučinić, Krpač, Mihoci; 15.VIII.2011. Currently deposited in coll. WG, will be deposited in the Croatian Natural History Museum, Zagreb, Croatia.

##### Paratypes.

3 males: Macedonia, Mavrovo National Park, Korab Mountains, Reč; leg. Krpač, Mihoci, Kučinić; 01.VIII.2011. Currently deposited in coll. MK, two paratypes will be deposited in the Macedonian Museum of Natural History, Skopje, Republic of Macedonia, one paratype will be deposited in coll. WG.

##### Type locality.

Macedonia, Korab Mountains.

##### Diagnosis.

Males of the new species are most similar to *Drusus macedonicus*, but exhibit (1) a distally straight ventral half of segment IX; (2) a dorsally straight tip of the intermediate appendage distinctly separated by a proximal indentation and with small proximal and distal rough protrusions; (3) a conical inferior appendage with a proximal dorsal triangular protrusion; (4) parameres with three tines in the distal third in dorsal view. *Drusus macedonicus* males have a distally concave ventral half of segment IX, intermediate appendages with two rough rounded dorsad protrusions but lacking a distinct proximal indentation, distally tapering inferior appendages, and parameres with a single tine in the distal third in dorsal view.

##### Description.

*Adults*. Habitus yellow; sternites and tergites fawn; cephalic and thoracic setal areas pale; cephalic and thoracic setation blond, abdominal setation scarce, blond; legs fawn; haustellum and intersegmental integument pale, whitish; wings yellow with blond setae on veins and the membrane. Male maxillary palp 3-segmented. Forewing length 11 mm, spur formula 1–3–3.

**Figure 1.**
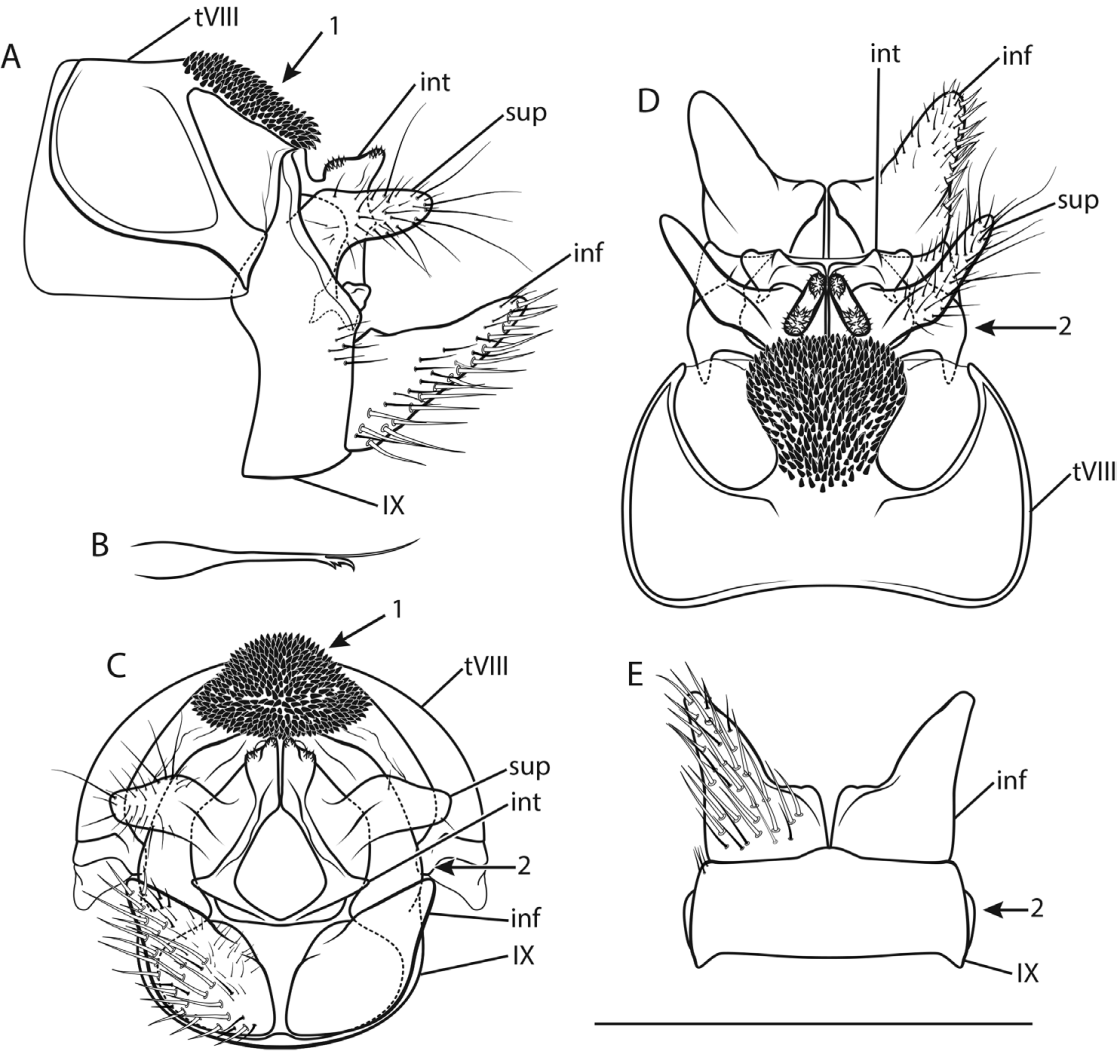
Male genitalia of *Drusus krpachi* sp. n. **A** left lateral view **B** paramere, dorsal view **C** caudal view **D** dorsal view **E** ventral view. Abbreviations: tVIII tergite VIII, IX segment IX, sup superior appendage, int intermediate appendage, inf inferior appendage; arrow 1 indicates spinose area of tergite VIII, arrow 2 indicates lateral protrusion of segment IX. Scale bar denotes 1 mm. Del. Vitecek.

*Male genitalia* (Fig. 1). Tergite VIII (tVIII) fawn, setae absent; spinose area in lateral view approximately flat with slight dorsocaudal protrusion in cranial half, in dorsal view suboval; flanked by membranous, less sclerotized areas. Segment IX (IX) in lateral view ventrally straight distally; in caudal view dorsally approximately as wide as ventrally; with rounded lateral protrusion in the dorsal half (best seen in ventral view). Superior appendages (sup) in lateral view suboval, curved obtusely caudad in proximal third, proximally with slight dorsal protrusion, longest in anterioposterior axis: approximately 2.5 times longer than high; in dorsal view proximally slightly concave medially; medial transverse section oval. Intermediate appendages (int) in lateral view with subtriangular tip, rough areas concentrated on dorsal proximal and dorsal distal aspect; in dorsal view tips separated, oval, distally converging; in caudal view approximately triangular. Inferior appendages (inf; gonopods *sensu* Snodgrass 1935) in lateral view conical, proximally wide, distally slender, with proximal triangular protrusion dorsally; in ventral and dorsal view with small medial projection and slight notch. Parameres simple, in dorsal view with 3 tines in distal third: 2 mediolateral, 1 dorsal.

Female and pupa unknown. Larval description and indentification key provided by Vitecek et al. (in press).

##### Etymology.

Named after V. Krpač, Macedonian entomologist and collector of the species.

##### Distribution.

Micro-endemic of the Korab Mountains, Hellenic Western Balkans (ecoregion 6, Illies 1978) (Fig. 11).

#### Drusus malickyi

Taxon classificationAnimaliaTrichopteraLimnephilidae

Oláh & Vitecek sp. n.

http://zoobank.org/4A7440AE-973D-4F33-858C-D53A48EEF743

Fig. 2

##### Material examined.

**Holotype.** 1 male: Albania, Shkoder County, Shkoder District, Prokletije Mts, beech forest with brook above Okol; N42.42258, E19.76127; leg. Puskas 05.IX.2013. Currently deposited in coll. WG, will be deposited in János Oláh Private Collection under national protection of the Hungarian Natural History Museum, Budapest, Hungary (JO).

##### Type locality.

Albania, Prokletije Mountains.

##### Diagnosis.

The holotype of the new species is most similar to *Drusus macedonicus*, but exhibits (1) a sharp mediocaudal protrusion of segment IX; (2) a dorsally straight and rough tip of the intermediate appendage distinctly separated by a proximal indentation (3) a distinctly slender and constricted distal half of the inferior appendage in lateral view. *Drusus macedonicus* males have a mediocaudal and a ventrocaudal protrusion of segment IX, intermediate appendages with two rough rounded dorsal protrusions but lacking a distinct proximal indentation, and to a lesser degree constricted inferior appendages.

##### Description.

*Adult, holotype*. Habitus yellow; sternites and tergites fawn; cephalic and thoracic setal areas pale; cephalic and thoracic setation blond, abdominal setation scarce, blond; legs fawn; haustellum and intersegmental integument pale, whitish; wings yellow with blond setae on veins and the membrane. Male maxillary palp 3-segmented, forewing length 10.9 mm, spur formula 1–3–3.

**Figure 2.**
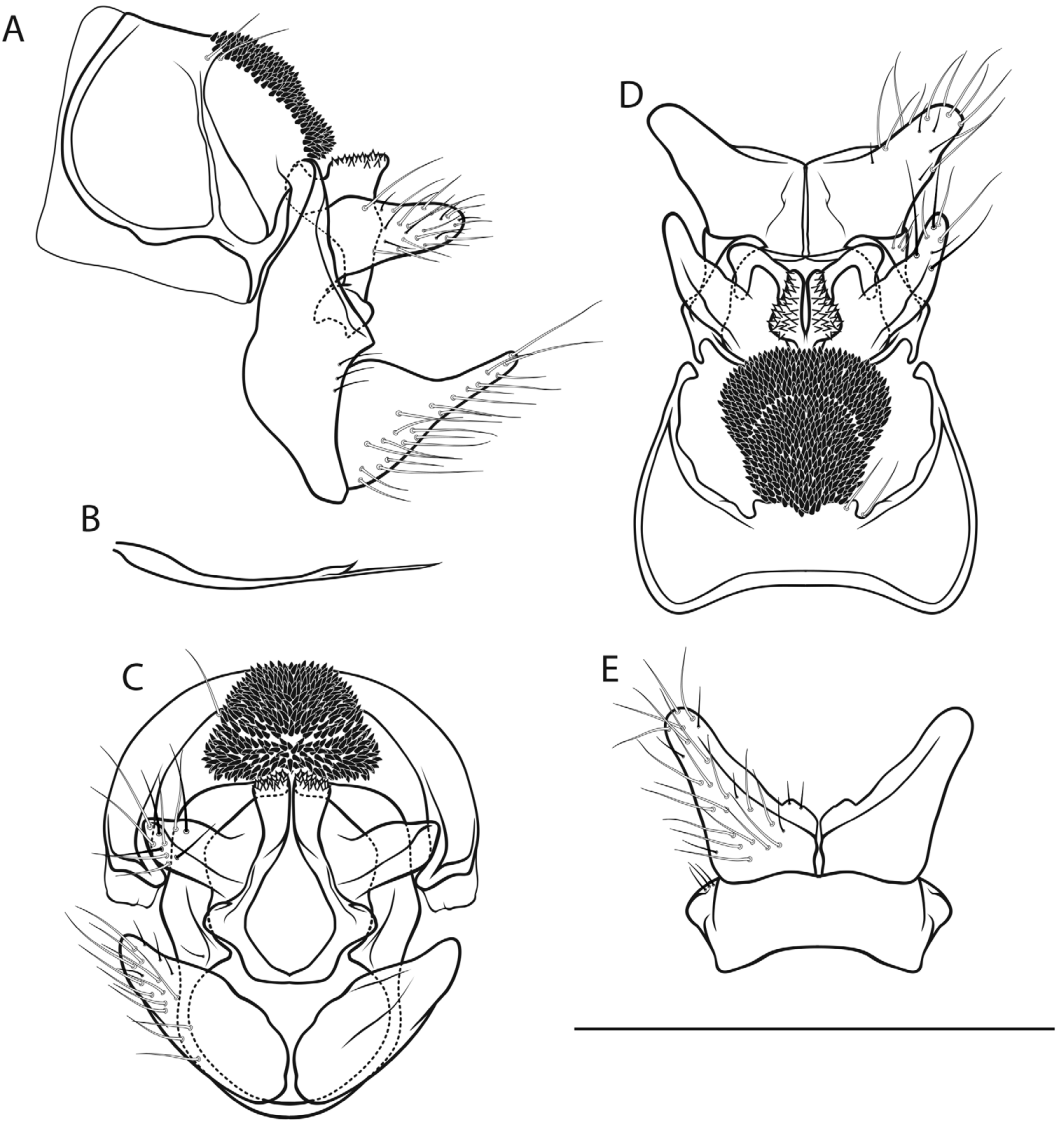
Male genitalia of *Drusus malickyi* sp. n. **A** left lateral view **B** paramere, lateral view **C** caudal view **D** dorsal view **E** ventral view. Scale bar denotes 1 mm. Del. Vitecek.

*Male genitalia* (Fig. 2). Tergite VIII fawn, setae scarce; spinate area in lateral view approximately flat with slight dorsad protrusion in anterior half, in dorsal view suboval; flanked by membraneous, less sclerotized areas. Segment IX in lateral view with sharp medial caudal protrusion, ventrally concave distally; in caudal view wider dorsally than ventrally; with irregular triangular, rounded lateral protrusion in dorsal half (best seen in dorsal and ventral views). Superior appendages in lateral view suboval, curved obtusely caudad in proximal quarter, proximally with slight dorsal and distinct ventral protrusions, longest in anterioposterior axis: approximately 2.5 times longer than high; in dorsal view proximally concave medially; medial transverse section oval. Intermediate appendages in lateral view with subtriangular, dorsally rough tip; in dorsal view tips separated, wedge-shaped, approximately parallel; in caudal view approximately triangular. Inferior appendages in lateral view subtriangular, proximally somewhat bulbous, distally slender and distinctly constricted; in ventral and dorsal views with small medial protrusion and slight notch; in ventral view with longitudinal groove delimiting medial lobe. Parameres simple, in lateral view with 1 tine in distal third.

Female, pupa and fifth instar larva unknown.

##### Etymology.

Named after Hans Malicky, trichopterologist.

##### Distribution.

Micro-endemic of the Prokletije Mountains, Hellenic Western Balkans (ecoregion 6) (Fig. 11).

### Re-descriptions of male filtering carnivore Drusinae *sensu* Pauls et al. (2008)

#### Cryptothrix nebulicola

Taxon classificationAnimaliaTrichopteraLimnephilidae

McLachlan, 1867

Fig. 3

##### Material examined.

1 male: Italy, Torino, Traversella, Fondo, Burdeivier brook; leg. Vincon; 12.VII.2012. 12 males: Italy, San Marco Pass; leg. Graf; 14.VIII.2000; in coll. WG.

##### Type locality.

Switzerland, Canton of Valais, Maienwang (Grimselpass).

##### Description.

*Adults.* Habitus dark; sternites and tergites brown; cephalic and thoracic setal areas pale; cephalic, thoracic and abdominal setation blond; legs light brown to fawn, proximally darker; haustellum and intersegmental integument pale, whitish; wings dark, with dark setae. Male maxillary palp 3-segmented; forewing length 8–10 mm; spur formula 1–2–2.

**Figure 3.**
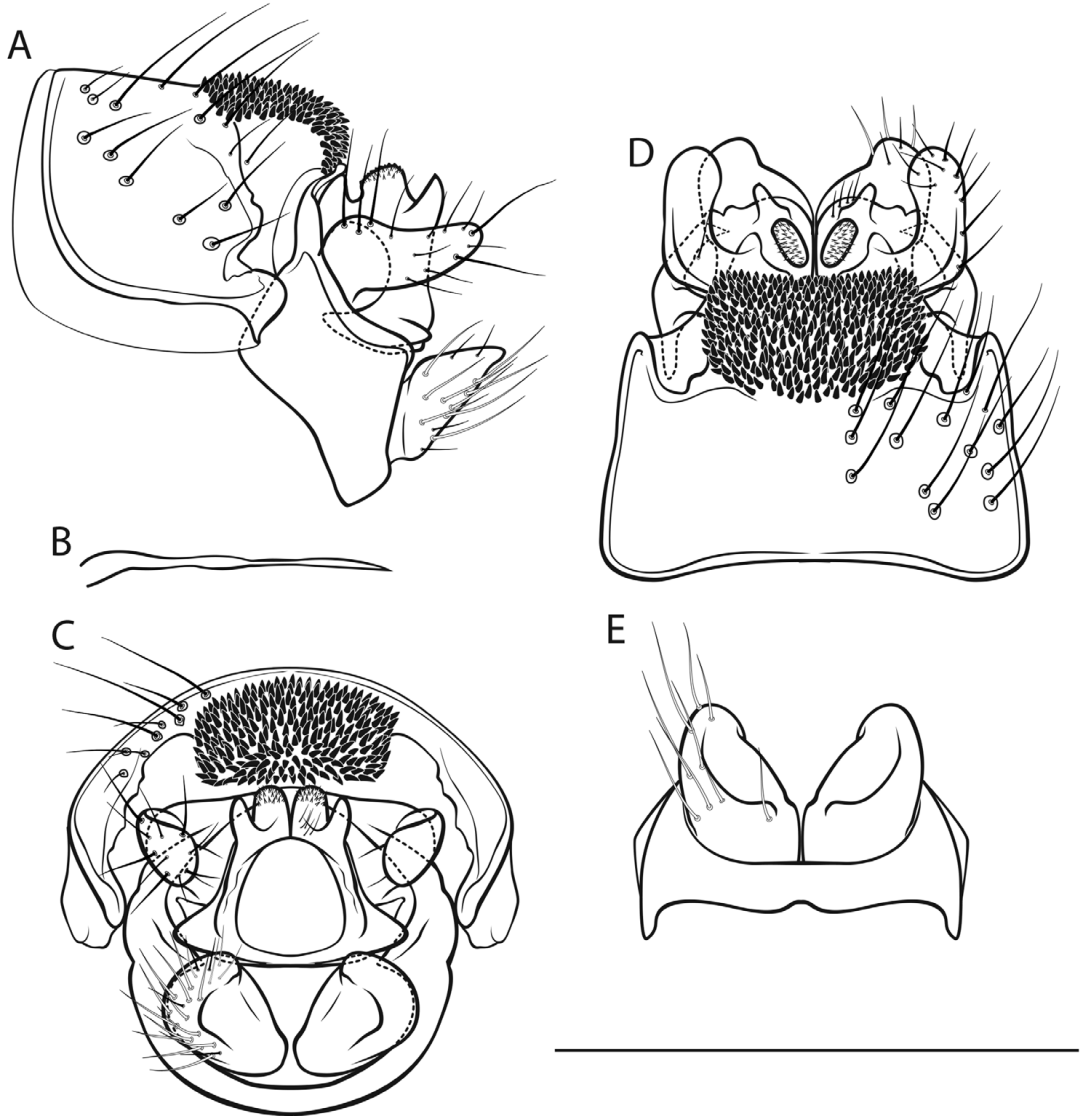
Male genitalia of *Cryptothrix nebulicola*. **A** left lateral view **B** paramere, lateral view **C** caudal view **D** dorsal view **E** ventral view. Scale bar denotes 1 mm. Del. Vitecek.

*Male genitalia* (Fig. 3). Tergite VIII brown, with lighter areas around alveoli; setation abundant; spinose area approximately rectangular in dorsal view; flanked by membraneous, less sclerotized areas. Segment IX in lateral view ventrally slightly concave distally; in caudal view wider dorsally than ventrally; with long, round, wedge-shaped protrusion in dorsal half (best seen in ventral view). Superior appendages in lateral view suboval, curved obtusely caudad in proximal fifth, proximally dorsal somewhat protuberant, tips slightly curved dorsad, longest in anterioposterior axis: approximately 2 times longer than high; in dorsal view medially concave, tips converging; medial transverse section lateroventrally curviconvex suboval. Intermediate appendages in lateral, dorsal and caudal views dorsally with 2 distinct tips, the proximal tip rounded, rough, the distal tip pointed, smooth; in caudal view approximately an isoceles trapezium. Inferior appendages in lateral view roughly triangular, proximally constricted, ventrocaudally slightly concave; in dorsal and ventral views tips converging; in ventral view with longitudinal groove delimiting medial lobe. Parameres simple, rodlike, medially and distally somewhat bulbous.

Female depicted by Schmid (1956), Malicky (2004); larva in key presented by Waringer and Graf (2011), Vitecek et al. (in press); pupa unknown.

##### Distribution.

Regionally in the Western Alps (ecoregion 4) (Fig. 11).

#### Drusus chrysotus

Taxon classificationAnimaliaTrichopteraLimnephilidae

Rambur, 1842

Fig. 4

##### Material examined.

12 males: Austria, Krumbach, Soboth; N46.723, E15.0555; leg. Graf; 20.V.2004; in coll. WG.

##### Type locality.

France, Rhône-Alpes, Haute-Savoie, Chamonix valley.

##### Description.

*Adults.* Habitus: light brown to yellow; sternites and tergites light brown, abdominal tergite VII with distinct saddle; cephalic and thoracic setal areas pale; cephalic and thoracic setation blond, abdominal setation scarce, short, dark; legs fawn, proximally darker; haustellum and intersegmental integument pale, whitish; wings light brown to yellow with dark setae on veins and blond setae on membrane. Male maxillary palp 3-segmented; forewing length 14–16 mm; spur formula 1–3–3.

**Figure 4.**
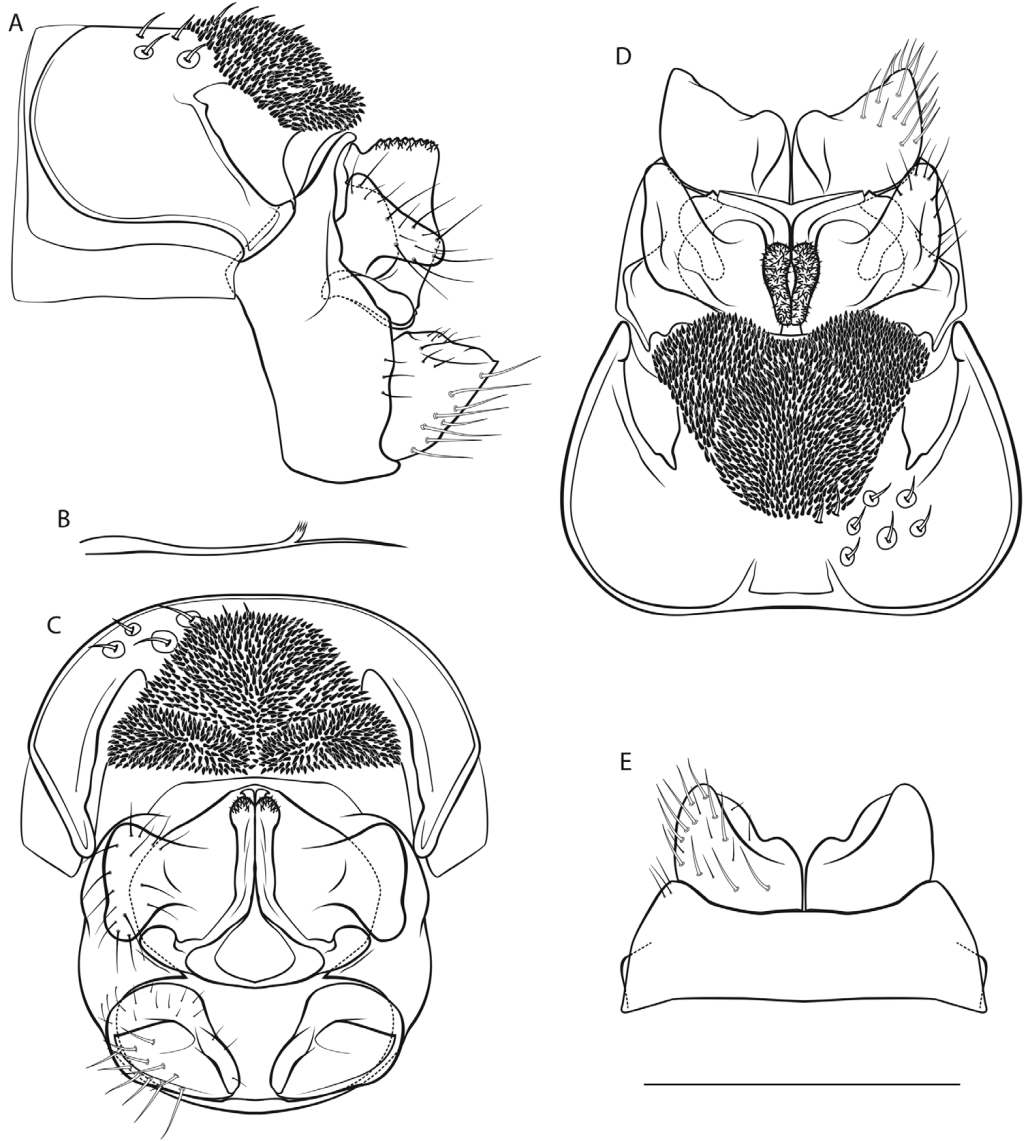
Male genitalia of *Drusus chrysotus*. **A** left lateral view **B** paramere, lateral view **C** caudal view **D** dorsal view **E** ventral view. Scale bar denotes 1 mm. Del. Vitecek.

*Male genitalia* (Fig. 4). Tergite VIII light brown, with short, pale, translucent setae; spinose area in lateral view with distinct dorsal protrusion and dorsomedial caudal protrusion, in dorsal and caudal views tripartite; flanked by membraneous, less sclerotized areas. Segment IX in lateral view ventrally irregular concave distally; dorsally approximately as wide as ventrally in caudal view; with distinct approximately subtriangular, rounded protrusion in dorsal half (best seen in dorsal and ventral views). Superior appendages in lateral view curved obtusely ventrocaudad in proximal third, proximally with distinct dorsocranial protrusion, approximately as long as high, in dorsal view proximally concave medially; medial transverse section oval. Intermediate appendages in lateral view medially protruding caudad, dorsally with long, rough tip; in dorsal view fused into approximately heart-shaped, centrally indented structure; in caudal view ventrally broad with bulbous lateral protrusions, dorsally narrow, fused. Inferior appendages in lateral view conical, short; in ventral and dorsal views blunt, with blunt, short medial protrusion and slight notch; in ventral view with longitudinal groove delimiting medial lobe. Parameres simple with several tines on common base in distal third.

Female depicted by Schmid (1956), Malicky (2004); larva in key presented by Waringer and Graf (2011), Vitecek et al. (in press); pupa described in Bohle (1987).

##### Distribution.

This species is widely distributed, occuring in and around the Alpine arc (ecoregion 4), the Western and Central Highlands (ecoregions 8 & 9) and was also found in the northern part of the Dinaric Alps (ecoregion 5) (Fig. 11).

#### Drusus discolor

Taxon classificationAnimaliaTrichopteraLimnephilidae

Rambur, 1842

Fig. 5

##### Material examined.

3 males: France, Mt. Canigou; N42.4864, E2.4139; leg. Graf; 12.VII.2012; in coll. WG. 2 males: France, St. Pierre de la Martin; N42.9597, E0.8290; leg. Graf; 22.VII.2012; in coll. WG. 7 males: Austria, Gurkursprung; leg. Wieser; 13.VII.1997; in coll. WG. 22 males: Switzerland, Val Munstair; N46.5852, E10.4544; leg. Graf; 20.VII.2006; in coll. WG. 1 male: Montenegro, Brodavac, right tributary of Peručica; N42.6859, E19.7364; leg. A. Previšić; 10.VII.2013; in coll. AP.

##### Type locality.

France, Rhône-Alpes, Haute-Savoie, Chamonix valley.

##### Description.

*Adults.* Habitus fawn to brown; sternites and tergites fawn to brown; cephalic and thoracic setal areas pale; cephalic, thoracic and abdominal setation blond; legs fawn, proximally darker; haustellum and intersegmental integument pale, whitish; wings blond-brown, with blond-brown setae on veins and blond setae on membrane. Male maxillary palp 3-segmented; forewing length 12–15 mm, spur formula 1–3–3.

**Figure 5.**
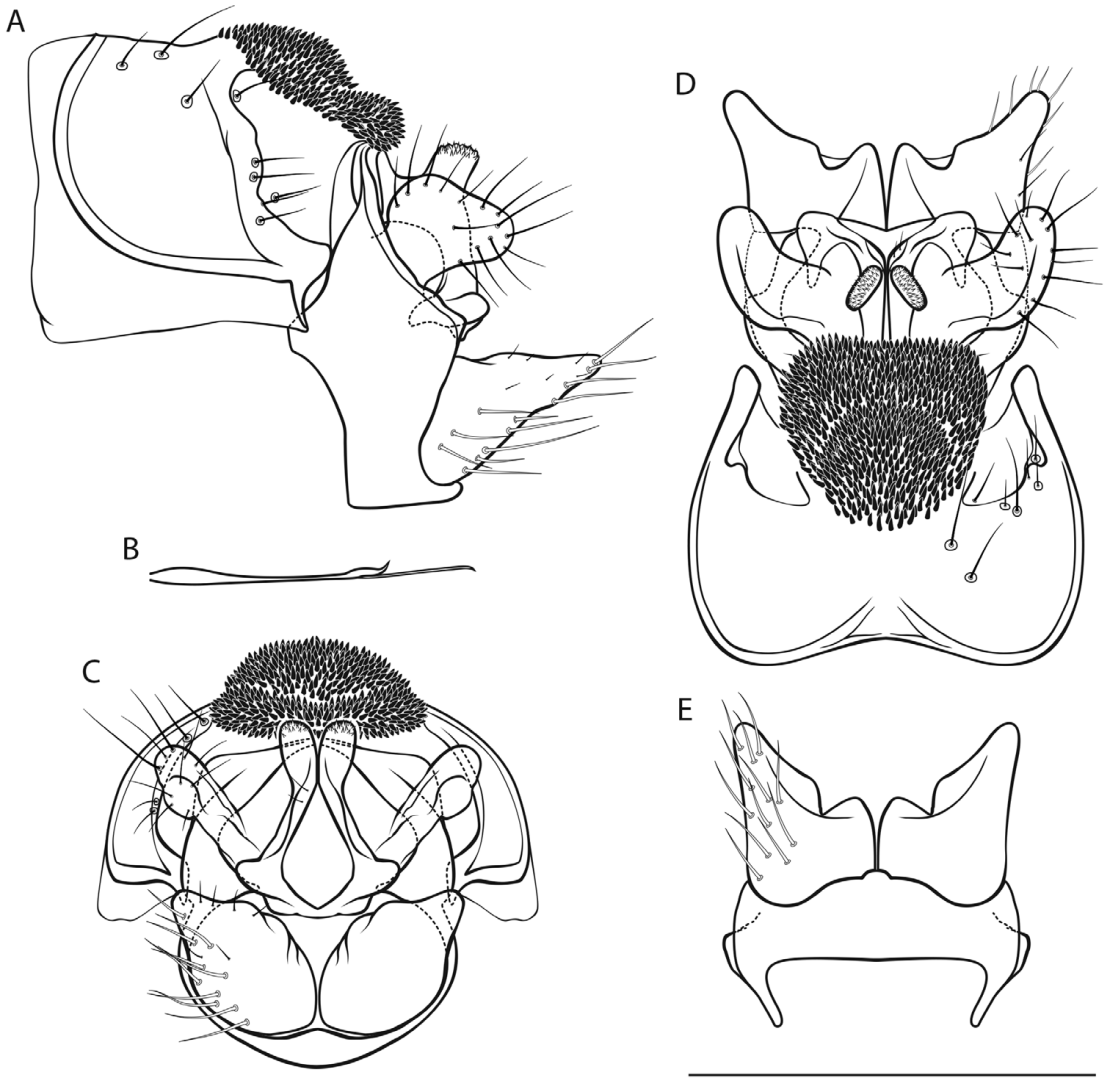
Male genitalia of *Drusus discolor*. **A** left lateral view **B** paramere, lateral view **C** caudal view **D** dorsal view **E** ventral view. Scale bar denotes 1 mm. Del. Vitecek.

*Male genitalia* (Fig. 5). Tergite VIII light brown, setation scarce, in lateral view with distinct cranial dorsal protuberance; spinose area in lateral view with distinct dorsal protrusion and dorsomedial caudal protrusion, in dorsal view suboval, caudally straight; flanked by membraneous, less sclerotized areas. Segment IX in lateral view ventrally distinctly concave distally; in caudal view dorsally approximately as wide as ventrally; with a distinct, caudally straight rounded protrusion indorsal half (best seen in ventral view). Superior appendages in lateral view suboval, curved obtusely caudad in proximal half, proximal half with distinct dorsal protrusion, approximately as long as high; in dorsal view medially concave; medial transverse section suboval. Intermediate appendages in lateral view medially approximately straight, dorsally with rounded, rough tip; in dorsal view tips separate, oval, distally converging; in caudal view approximately triangular with dorsally diverging tips. Inferior appendages in lateral view conical; in ventral and dorsal views with distinct medial protrusion and distinct notch; in ventral view with longitudinal groove delimiting medial lobe. Parameres simple with single bulbously based tine in distal third.

Female depicted by Schmid (1956), Malicky (2004); larva in key presented by Waringer and Graf (2011), Vitecek et al. (in press); pupa unknown.

##### Distribution.

This species is one of the most widespread Drusinae species, covering all major European mountain ranges from the Carpathians to the Pyrenees (ecoregions 1–10) (Fig. 11).

#### Drusus macedonicus

Taxon classificationAnimaliaTrichopteraLimnephilidae

Schmid, 1956

Fig. 6

##### Material examined.

1 male: Macedonia, Jablanica Mt., Labunište; N41.271841, E20.558136; leg. Kučinić and Krpač; 19.IX.2013; in coll. MK. 1 male: Macedonia, Pelister Mt., springs of Caparska reka; N41.003889, E21.167944; leg. Graf and Previšić; 07.VII.2010; in coll. WG.

##### Type locality.

Macedonia, Pelister Mountains.

##### Description.

*Adults.* Habitus yellow; sternites and tergites fawn; cephalic and thoracic setal areas pale; cephalic and thoracic setation blond, abdominal setation scarce, blond; legs fawn; haustellum and intersegmental integument pale, whitish; wings yellow with blond setae on veins and membrane. Male maxillary palp 3-segmented; forewing length 10–12 mm; spur formula 1–3–3.

**Figure 6.**
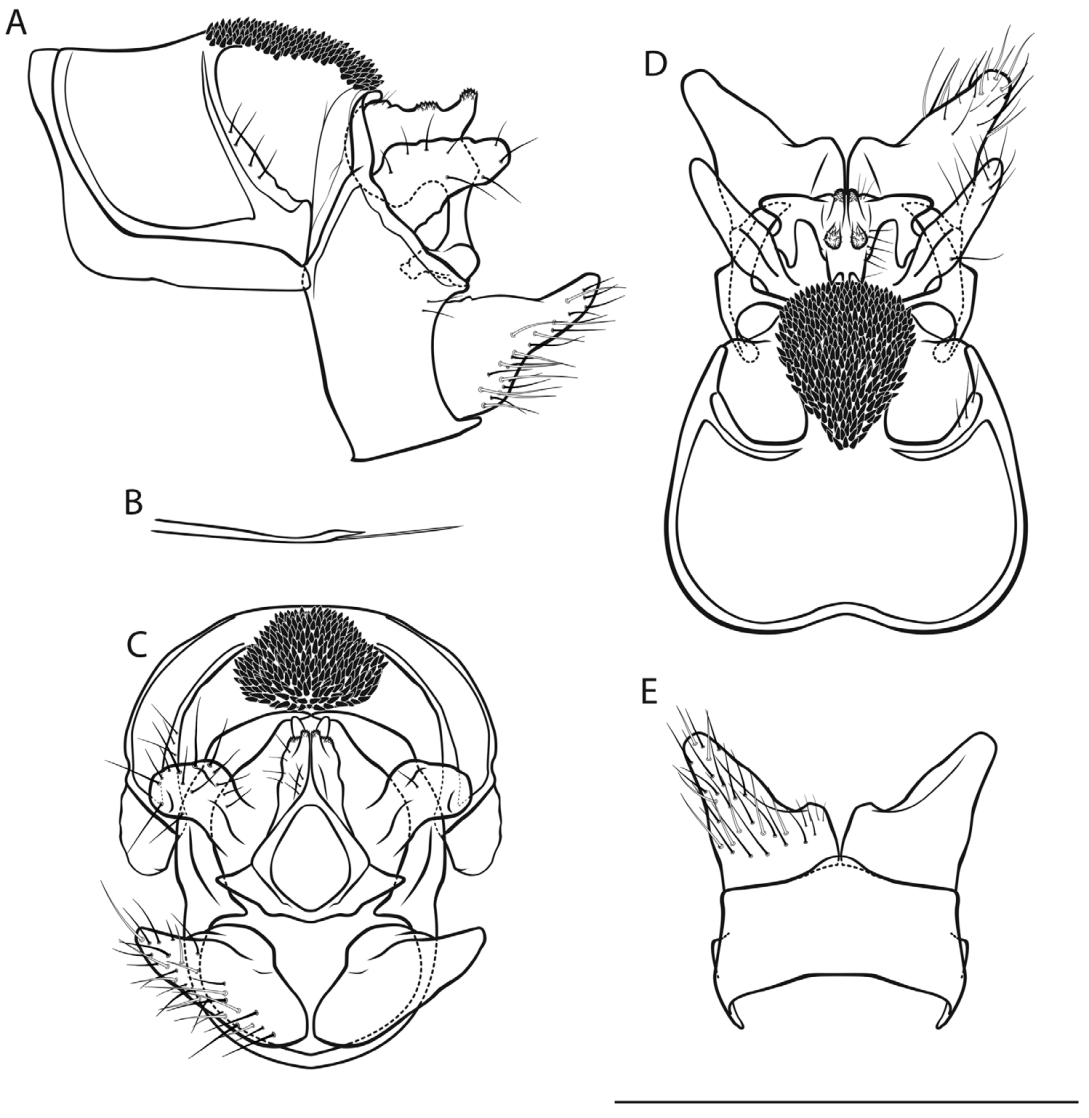
Male genitalia of *Drusus macedonicus*. **A** left lateral view **B** paramere, lateral view **C** caudal view **D** dorsal view **E** ventral view. Scale bar denotes 1 mm. Del. Vitecek.

*Male genitalia* (Fig. 6). Tergite VIII fawn, setation lateral, scarce; spinose area in lateral view approximately flat, in dorsal view suboval, tapering cranially; flanked by membraneous, less sclerotized areas. Segment IX in lateral view ventrally deeply concave distally, with distinct medial and ventral caudad protrusion; in caudal view slightly wider dorsally than ventrally; with sharp, caudally approximately straight protrusion in dorsal half (best seen in dorsal and ventral views). Superior appendages in lateral view irregularly suboval, curved obtusely caudad in proximal quarter, proximally with an irregular dorsal and irregular ventral protuberance, longest in anterioposterior axis: approximately 2.5 times longer than high; in dorsal view proximally slightly concave medially; medial transverse section suboval. Intermediate appendages in lateral view with two rough tips: 1 curved dorso-posteriorly, 1 central, rounded; in dorsal view posterior tips adjacent, parallel; in caudal view subtriangular with slender lateral projections. Inferior appendages in lateral view approximately conical, proximally wide, distally slender; in ventral and dorsal views with medial tip and notch, separated by slight notch; in ventral view with longitudinal groove delimiting medial lobe. Parameres simple, with single dorsal tine in distal third.

Female depicted by Schmid (1956), Malicky (2004); larva in key presented by Vitecek et al. (in press); pupa unknown.

##### Distribution.

Micro-endemic of the Pelister and Jablanica Mountains, Hellenic Western Balkans (ecoregion 6) (Fig. 11).

#### Drusus meridionalis

Taxon classificationAnimaliaTrichopteraLimnephilidae

Kumanski, 1973

Fig. 7

##### Material examined.

10 males: Bulgaria, Vihren, Pirin Mountains, Okotovo-Banserishka, marshy spring; N41.7389, E23.4462; leg. Keresztes, Török, Kolcsár; 23.VIII.2013; in coll WG.

##### Type locality.

Bulgaria, Rila and Pirin Mountains.

##### Description.

*Adults.* Habitus yellow to brown; sternites and tergites yellow to brown; cephalic and thoracic setal areas pale; cephlic and thoracic setation blond, abdominal setation scarce, short, dark; legs yellow to light brown, proximally darker; haustellum and intersegmental integument pale, whitish; wings yellow to fawn, with blond setae on veins and membrane. Male maxillary palp 3-segmented. Forewing length 12–14 mm; spur formula 1–3–3.

**Figure 7.**
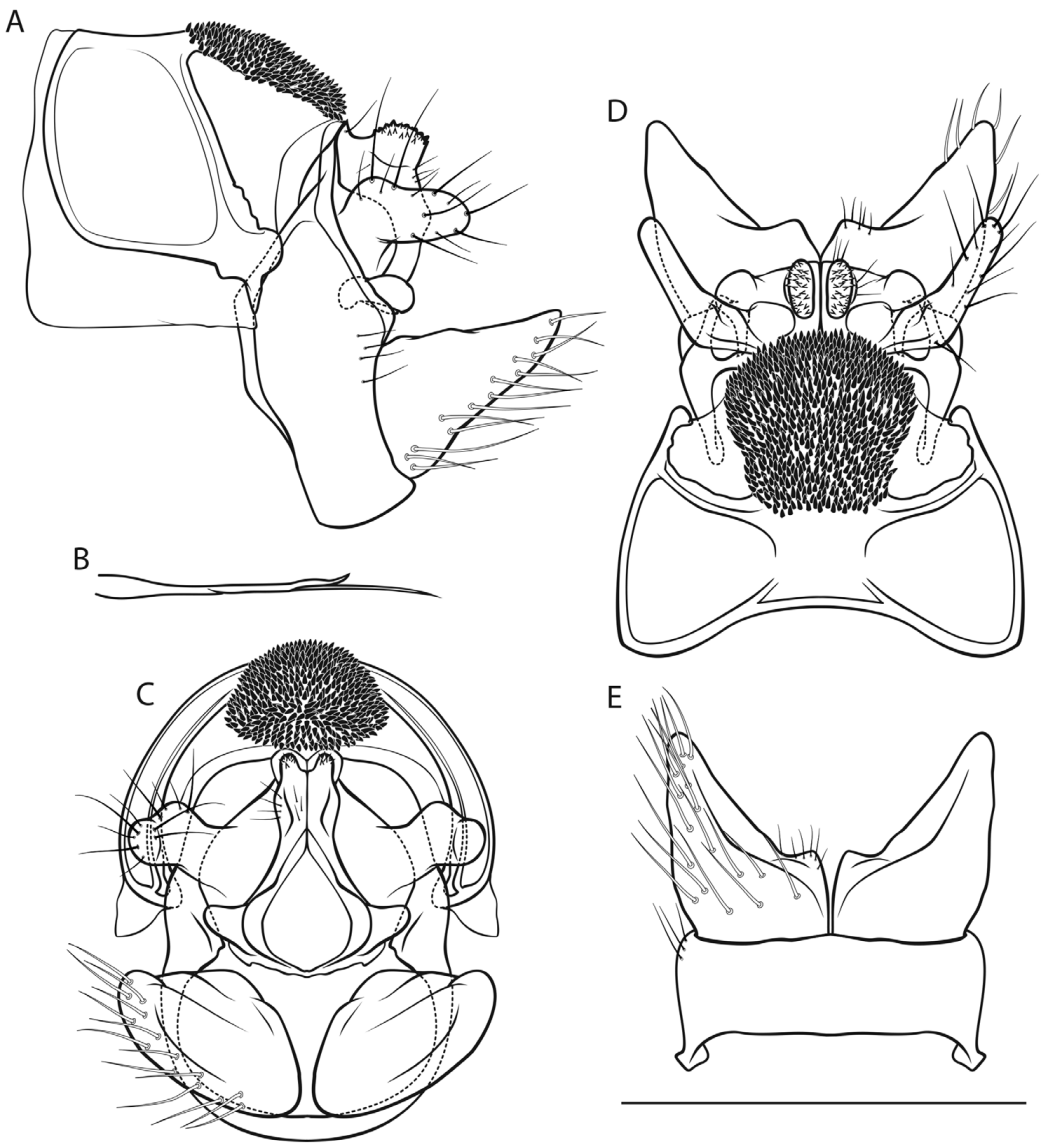
Male genitalia of *Drusus meridionalis*. **A** left lateral view **B** paramere, lateral view **C** caudal view **D** dorsal view **E** ventral view. Scale bar denotes 1 mm. Del. Vitecek.

*Male genitalia* (Fig. 7). Tergite VIII yellow to brown, setae absent; spinose area in lateral view approximately flat, in dorsal view suboval, somewhat rectangular cranially; flanked by membraneous, less sclerotized area bearing single seta. Segment IX in lateral view ventrally slightly concave distally; in caudal view wider ventrally than dorsally; with distinct, approximately triangular, rounded protrusion in dorsal half (best seen in dorsal view). Superior appendages in lateral view suboval, curved obtusely caudad in proximal third, proximally with distinct dorsal protrusion, longest in anterioposterior axis: approximately 3 times longer than high; in dorsal view proximally slightly concave medially; medial transverse section oval. Intermediate appendages in lateral view with rounded, rough tip; in dorsal view 2 separate parallel tips, each oval, rough; in caudal view subtriangular, dorsally with 2 separate tips. Inferior appendages in lateral view conical; in ventral and dorsal views slender with minute subtriangular medial protrusion and shallow notch; in ventral view with longitudinal groove delimiting medial lobe. Parameres simple, with single, bulbously based tine in distal third.

Female depicted by Kumanski (1973), Malicky (2004); larva in key presented by Vitecek et al. (in press); pupa unknown.

##### Distribution.

Regionally in the Eastern Balkans (ecoregion 7) (Fig. 11).

##### Comments.

This species was first described as a subspecies of *Drusus romanicus*, but was elevated to species rank (Vitecek et al. in press). It is morphologically distinct from *Drusus romanicus*, disjunct in distribution, and was recovered as a well-separated clade from *Drusus romanicus* in the phylogenetic analyses of Pauls et al. (2009) and Vitecek et al. (in press).

#### Drusus muelleri

Taxon classificationAnimaliaTrichopteraLimnephilidae

McLachlan, 1868

Fig. 8

##### Material examined.

1 male: Switzerland, Furkapass; N46.5888, E8.4327; leg. Graf; 21.VII.2006, in coll. WG.

##### Type locality.

Switzerland, Canton of Uri, Hospental.

##### Description.

*Adults.* Habitus dark; sternites and tergites brown; cephalic and thoracic setal areas pale, cephalic and thoracic setation blond, abdominal setation scarce, short, dark; coxa, trochanter, femur brown, tibia and tarsi fawn; haustellum and intersegmental integument pale, whitish; wings brown, smoky, with dark setae on veins and blond setae on membrane. Male maxillary palp 3-segmented; Forewing length 11–13 mm (Malicky 2004); spur formula 1–3–3.

**Figure 8.**
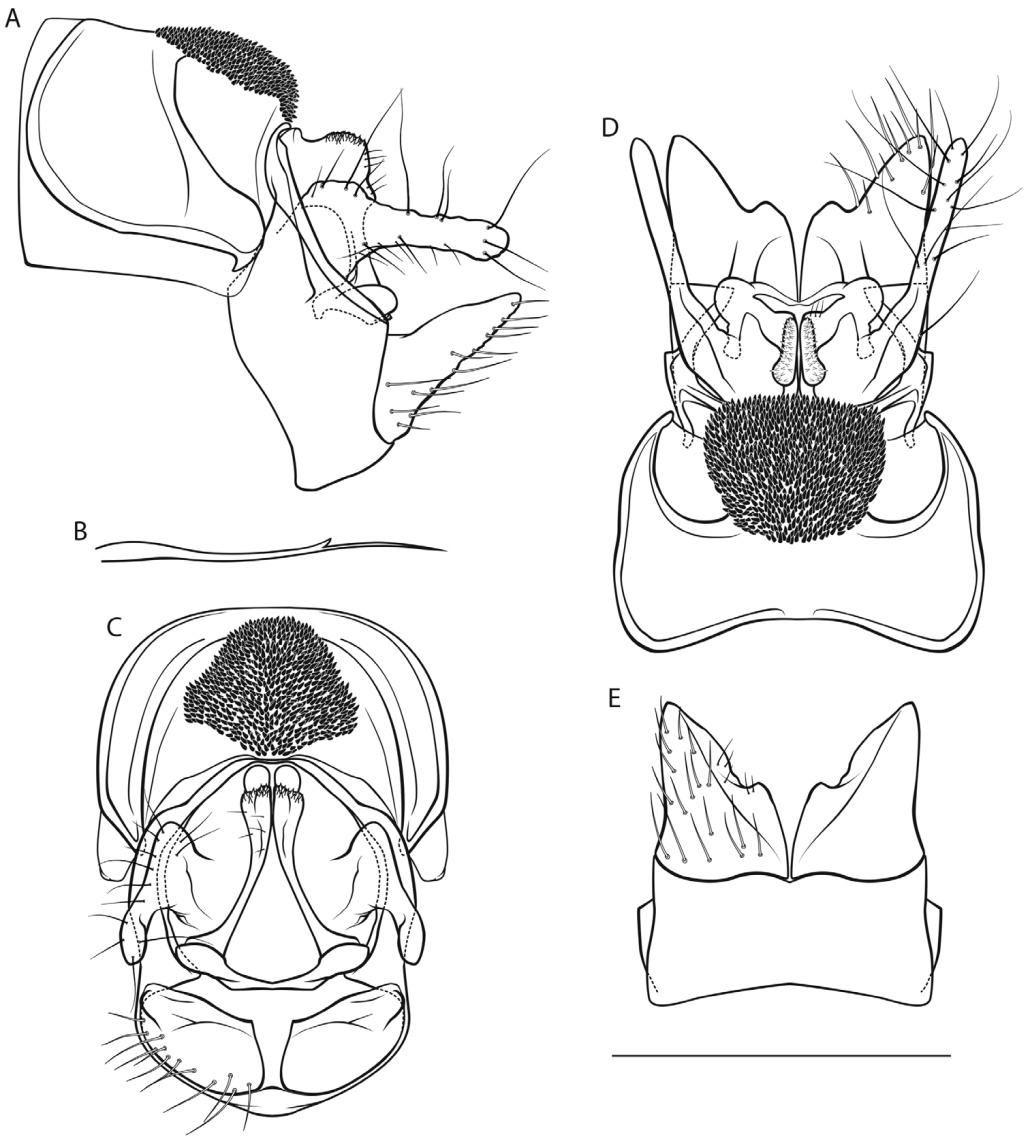
Male genitalia of *Drusus muelleri*. **A** left lateral view **B** paramere, lateral view **C** caudal view **D** dorsal view **E** ventral view. Scale bar denotes 1 mm. Del. Vitecek.

*Male genitalia* (Fig. 8). Tergite VIII brown, setae absent; spinose area in lateral view convex with caudal ventral lobe, in dorsal view suboval with small medial protrusion; flanked by membraneous, less sclerotized areas. Segment IX in lateral view ventrally slightly concave distally; in caudal view wider dorsally than ventrally; with sharp caudally straight subtriangular protrusion in the dorsal half (best seen in dorsal and ventral views). Superior appendages in lateral view irregular, curved obtusely caudad in proximal quarter, proximally distinctly dilated, distally clavate, longest in anterioposterior axis: approximately 5 times longer than high; in dorsal view the proximal third concave medially; medial transverse section circular. Intermediate appendages in lateral view with long, rounded rough tip; in dorsal view tips separate, approximately parallel, proximally bulbous; in caudal view subtriangular. Inferior appendages in lateral view subtriangular, ventrally irregular, proximally sightly concave dorsally; in ventral and dorsal views broad, with small subtriangular medial protrusion and distinct notch; in ventral view with longitudinal groove delimiting medial lobe; in caudal view broad. Parameres simple, with single dorsal tine in distal third.

Female depicted by Schmid (1956), Malicky (2004), larva in key presented by Waringer and Graf (2011), Vitecek et al. (in press); pupa unknown.

##### Distribution.

Regionally in the Western Alps (ecoregion 4) (Fig. 11).

#### Drusus romanicus

Taxon classificationAnimaliaTrichopteraLimnephilidae

Murgoci and Botosaneanu, 1953

Fig. 9

##### Material examined.

1 male: Romania, Apuseni Mts., Garda de Sus, tributary of Ariesul Mare; N46.4508, E22.7982; leg. Oláh, Bajka, Balogh, Borics; 29.V.2013; in coll. WG. 1 male: Romania, Apuseni Mts., Muntii Giaului, Stiunea Muntele Baisorii, Lupinus stream; leg. Oláh, Balogh, Fekete; 18.VI.2013; in coll. WG. 1 male: Romania, Retezat Mts, Bucara Stream, 150 m below Bucara lake; N45.3570, E22.8753; leg. Bajka, Balogh, Borics, Borics; 10.VIII.2013; in coll. WG.

##### Type locality.

Romania, Carpathian Mountains, spring areas of the Ialomita stream.

**Description.** *Adults.* Habitus brown to light brown; sternites and tergites brown to light brown; cephalic and thoracic setal areas pale; cephalic, thoracic and abdominal setation blond; legs light brown, proximally darker; haustellum and intersegmental integument pale, whitish; wings brown, proximally lighter, with blond setae on veins and membrane. Male maxillary palp 3-segmented; forewing length 12–14 mm; spur formula 1–3–3.

**Figure 9.**
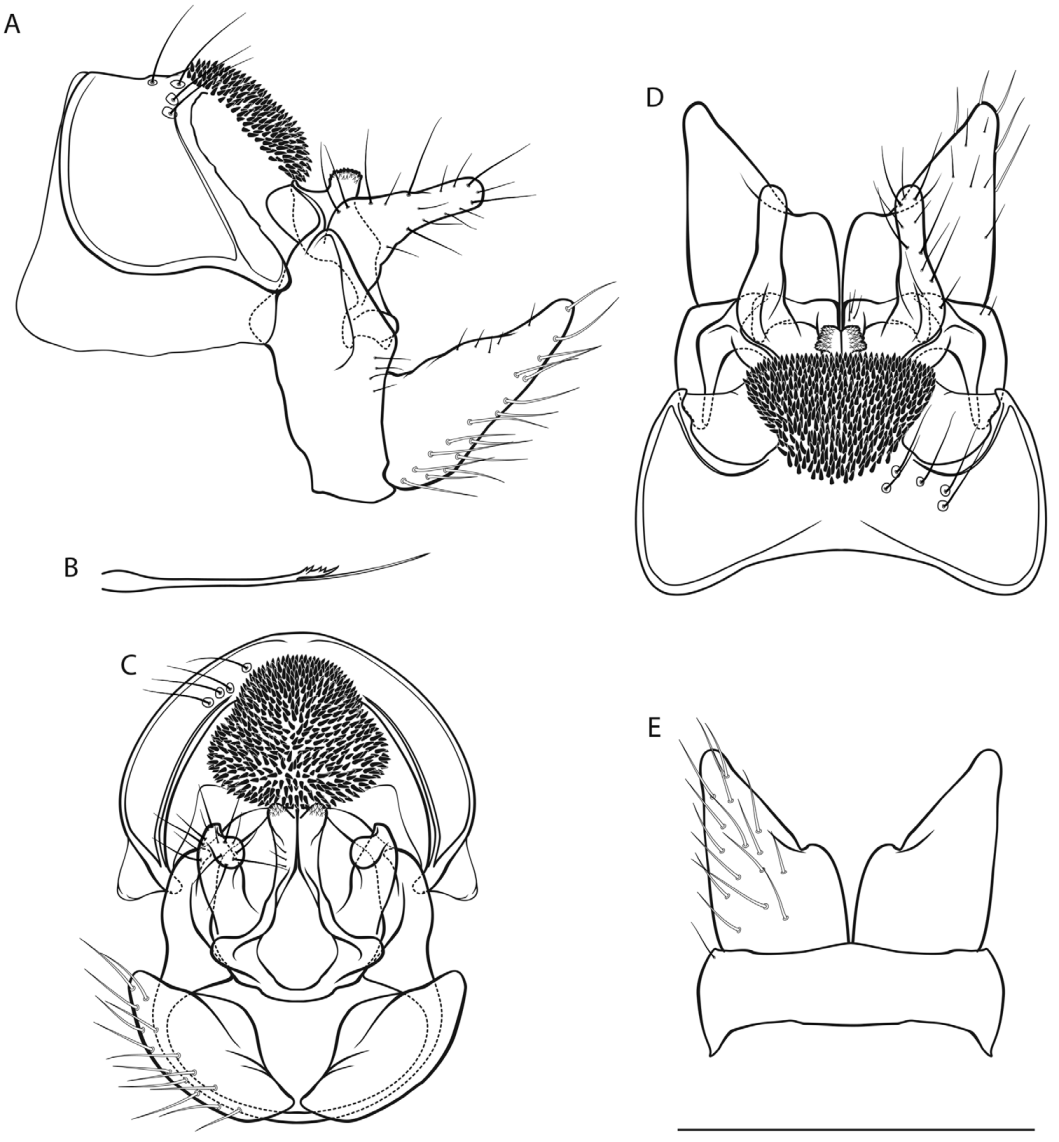
Male genitalia of *Drusus romanicus*. **A** left lateral view **B** paramere, lateral view **C** caudal view **D** dorsal view **E** ventral view. Scale bar denotes 1 mm. Del. Vitecek.

*Male genitalia* (Fig. 9). Tergite VIII brown, setae present; spinose area in lateral view approximately flat with slight dorsal protrusion, in dorsal view suboval, distally straight; flanked by membraneous, less sclerotized areas. Segment IX in lateral view dorsally with distinct notch distally, ventrally irregularly concave distally; in caudal view ventrally wider than dorsally; with distinct subtriangular rounded protrusion in dorsal half (best seen in dorsal view). Superior appendages in lateral view elongate suboval, curved obtusely dorsocaudad in proximal quarter, proximally with round dorsal protrusion and irregular ventral protrusion, longest in anterioposterior axis: approximately 4.5 times longer than high; in dorsal view proximally distinctly concave medially; medial transverse section circular. Intermediate appendages in lateral view with rounded, rough tip; in dorsal view tips separate, laterally diverging; in caudal view subtriangular. Inferior appendages in lateral view conical, long, dorsally irregular, proximally slightly concave dorsally; in ventral and dorsal views proximal half robust, distal half slender with slight medial protrusion and shallow notch. Parameres simple, with medial hook-shaped tip bearing several smaller tines.

Female and pupa unknown; larva in key presented by Vitecek et al. (in press).

##### Distribution.

Regionally in the Western and Southern Carpathians (ecoregion 10) (Fig. 11).

#### Drusus siveci

Taxon classificationAnimaliaTrichopteraLimnephilidae

Malicky, 1981

Fig. 10

##### Material examined.

5 males: Bosnia and Herzegovina, Sutjeska National Park, stream close to Čermerno; N43.2650, E18.5927; leg. Previšić, Miliša; 04.VII.2012; in coll. AP.

##### Type locality.

Montenegro, Andrijevica, Gnjili Potok.

##### Description.

*Adults.* Habitus yellow to fawn; sternites and tergites fawn; cephalic and thoracic setal areas pale; cephalic, thoracic and abdominal setation blond; legs yellow to fawn; haustellum and intersegmental integument pale, whitish; wings fawn, with blond to brown setae on veins and blond setae on membrane. Male maxillary palp 3-segmented, forewing length 10–12 mm, spur formula 1–3–3.

**Figure 10.**
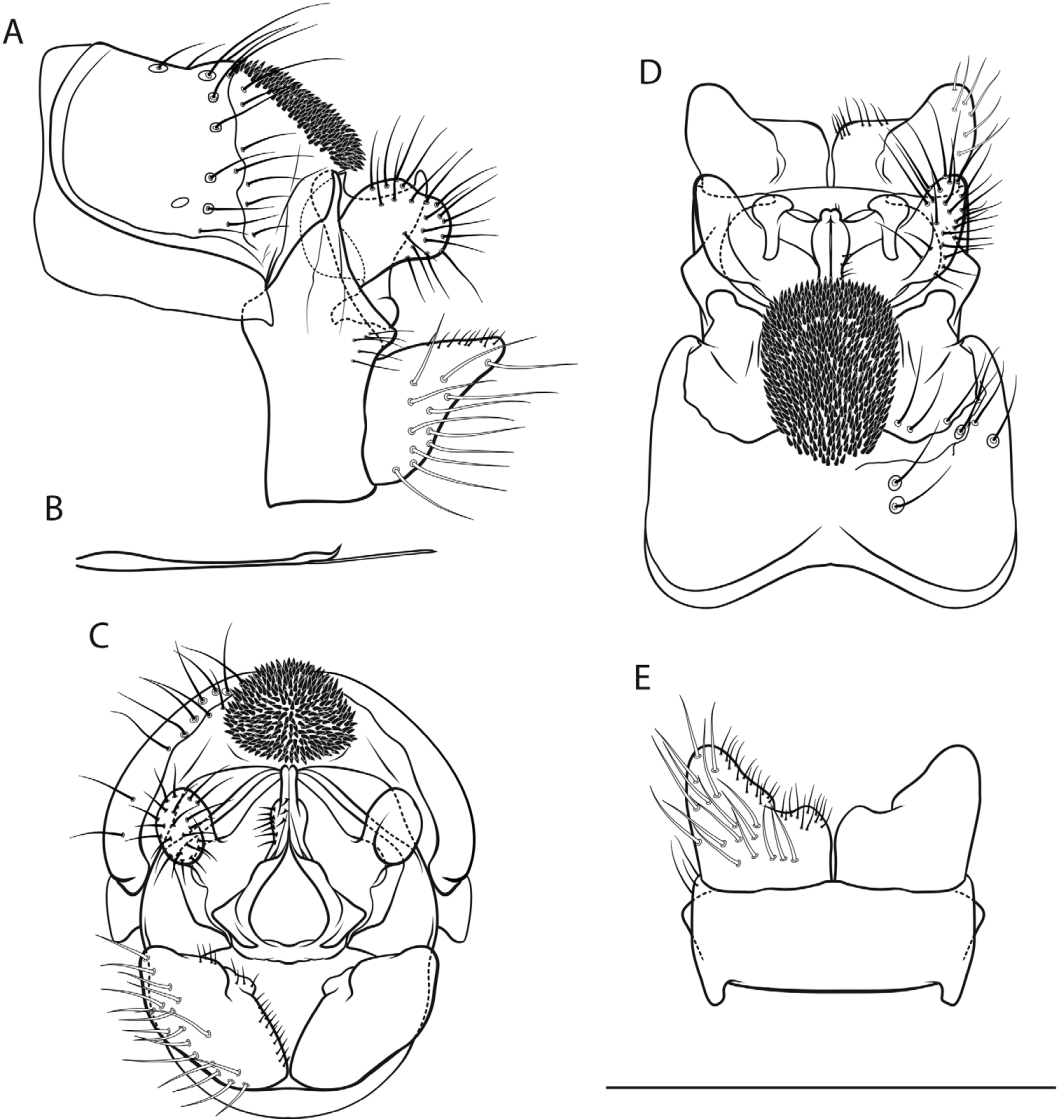
Male genitalia of *Drusus siveci*. **A** left lateral view **B** paramere, lateral view **C** caudal view **D** dorsal view **E** ventral view. Scale bar denotes 1 mm. Del. Vitecek.

*Male genitalia* (Fig. 10). Tergite VIII fawn, setation concentrated dorsally and posterolaterally, with slight dorsal protrusion; spinose area in lateral view approximately flat, in dorsal view oval; flanked by membraneous, less sclerotized areas. Segment IX in lateral view with medial caudad protrusion, ventrally irregularly concave distally; in caudal view wider dorsally than ventrally; with distinct rounded triangular protrusion in dorsal half (best seen in dorsal and ventral views). Superior appendages in lateral view suboval, curved obtusely caudad in proximal half, proximal half with distinct rounded protrusion, in dorsal view slightly concave medially; medial transverse section subcircular. Intermediate appendages in lateral view with pointed, hook-like tip arching dorsad; in dorsal and caudal views the tips fused; in caudal view subtriangular. Inferior appendages in lateral view conical, short, blunt, posteroventrally somewhat concave; in ventral view with medial protrusion and distinct notch. Parameres simple, with single bulbously based tine in distal third.

Female and pupa unknown; larva in key presented by Vitecek et al. (in press).

##### Distribution.

**Figure 11.**
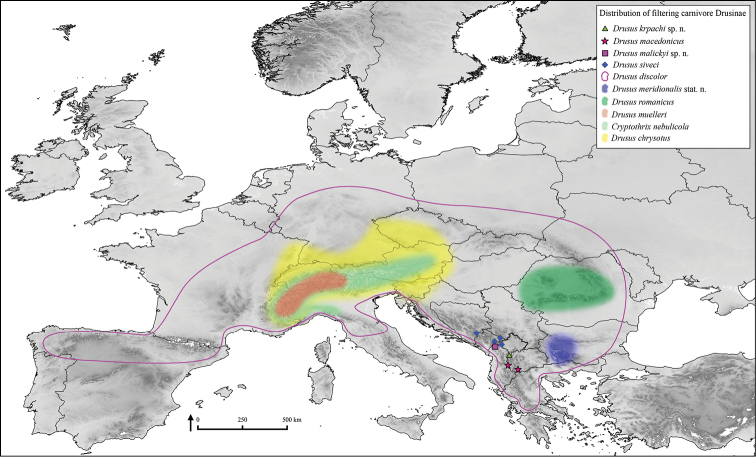
Distribution of filtering carnivore Drusinae. Single records of endemic species are depicted as symbols, stroked or filled areas denote ranges of more widely distributed species with a higher number of occurrence records.

Micro-endemic of the Dinaric Western Balkans (ecoregion 5) (Fig. 11).

## Discussion

### Drusinae micro-endemics of the Western Balkans

Morphology of the new species as well as molecular phylogenetic analyses (Vitecek et al. in press) suggest that the new species belong to the monophyletic clade of filtering carnivorous Drusinae *sensu* Pauls et al. (2008), comprising *Drusus discolor*, *Drusus muelleri*, *Drusus chrysotus*, *Drusus siveci*, *Drusus romanicus*, *Drusus meridionalis* and *Cryptothrix nebulicola*. The *Drusus* species described here are similar to *Drusus macedonicus* Schmid. However, they differ distinctly in the morphology of the male genitalia, particularly the intermediate appendages, and are discretely distributed. Also, they are highly supported in phylogenetic analysis and form a highly supported clade comprising (*Drusus malickyi* + (*Drusus krpachi* + *Drusus macedonicus*) in the phylogenetic analysis of Vitecek et al. (in press). To our knowledge, the new species are small-scale endemics restricted to single mountain ranges.

Interestingly, the type localities of the new species are close to the known range of *Drusus macedonicus* (Fig. 11). Such small-scale distribution of distinct Drusinae species is well documented from the Western Balkans (Marinković-Gospodnetić 1976; Kučinić et al. 2011a; Oláh 2010, 2011; Oláh and Kovács 2013; Previšić et al. 2014a, b; Vitecek et al. 2015). Similarly, other taxa exhibit comparable distribution patterns, in which single mountain ranges represent the range of a species, or haplogroups (Ursenbacher et al. 2008, Stevanović et al. 2009, Zogaris et al. 2009, Karaman et al. 2011). The intriguing distribution patterns exhibited by some groups of species potentially result from the geological history of the region and historic and present-day climate conditions. Small-scale speciation of Drusinae presumably is facilitated by intrinsic traits of the subfamily, such as their occurrence at higher elevations (Pauls et al. 2006, 2009), a putatively low long-distance dispersal potential (Müller-Peddinghaus 2011, Geismar et al. 2015), and might be further enhanced by habitat fragmentation, e.g., by regional karstification. Occurrence of Drusinae could therefore serve as proxy to occurrence of other aquatic invertebrate taxa, particularly to crenobiont taxa exhibiting the same or similar traits.

### Western Balkan aquatic diversity

The Western Balkans represent a hot-spot of species richness and endemicity in Europe (Griffiths et al. 2004, Guéorguiev 2007, Kenyeres et al. 2009, Jaskuła 2011). In particular, the faunas of isolated habitats such as coldwater springs and streams, caves or the profundal zone of large lakes contribute to high species richness in the region (Petkovski et al. 2009, Wilke et al. 2010, Pešić and Glöer 2013). Such taxa probably are more susceptible to factors promoting speciation, such as climatic and geological processes (e.g., karstification, see Previšić et al. 2009, 2014b), especially if their dispersal potential is low.

The description of the two new micro-endemic *Drusus* species increases the number of Western Balkan Drusinae species. Drusinae richness in the Western Balkans currently comprises 40 species including 13 species (30 %) that were discovered since 2010, of which 32 are endemic to the Western Balkans (Graf et al. 2008; Oláh 2010, 2011; Schmidt-Kloiber and Hering 2012; Oláh and Kovács 2013; Previšić et al. 2014b; Vitecek et al. 2015; Ibrahimi et al. pers. comm.; this study).

Thus, endemism rates of Western Balkan Drusinae are high, and are further augmented by the description of the two new micro-endemic *Drusus* species. Global and anthropogenic habitat changes are among the greatest threats to micro-endemic and endemic freshwater species (Hering et al. 2009, Tierno de Figueroa et al. 2010, Bálint et al. 2011, Conti et al. 2014). Water extraction for human consumption intensified by tourism, agriculture, and hydroelectricity are the primary modes of global anthropogenic habitat modification of freshwaters (Foster 1991, Polhemus 1993, Dudgeon 2006). Hydropower plants were identified as the greatest threat to European freshwater biodiversity (Freyhof 2012, Schwarz 2012, Zarfl et al. 2014, http://riverwatch.eu).

Recent published taxonomic works treating the Western Balkans, including the present one describing two new micro-endemic *Drusus* species, have demonstrated the significance of the region for European biodiversity. However, progressing socio-economic change and anthropogenic habitat modification threaten the freshwater biodiversity of the Western Balkans, and potentially will result in the loss of yet-to-be discovered species.

## Supplementary Material

XML Treatment for Drusus krpachi

XML Treatment for Drusus malickyi

XML Treatment for Cryptothrix nebulicola

XML Treatment for Drusus chrysotus

XML Treatment for Drusus discolor

XML Treatment for Drusus macedonicus

XML Treatment for Drusus meridionalis

XML Treatment for Drusus muelleri

XML Treatment for Drusus romanicus

XML Treatment for Drusus siveci

## References

[B1] Bálint M Domisch S Engelhardt CHM Haase P Lehrian S Sauer J Theissinger K Pauls SU Nowak C (2011) Cryptic biodiversity loss linked to global climate change. Nature Climate Change 1: 313–318. doi: 10.1038/nclimate1191

[B2] Bănărescu PM (2004) Distribution pattern of the aquatic fauna of the Balkan peninsula. In: Griffiths HI Kryštufek B Reed J (Eds) Balkan Biodiversity. Kluwer Academic Publishers, Dordrecht, 203–217. doi: 10.1007/978-1-4020-2854-0_12

[B3] Bianco PG (1998) Diversity of Barbinae fishes in southern Europe with description of a new genus and a new species (Cyprinidae). Italian Journal of Zoology 65(S1): 125–136. doi: 10.1080/11250009809386804

[B4] Bohle HW (1987) Drift-fangende Köcherfliegen-Larven unter den Drusinae (Trichoptera: Limnephilidae). Entomologia generalis 12(12-3): 119–132.

[B5] Bohlen J Perdices A Doadrio I Ecnomidis PS (2006) Vicariance, colonisation, and fast local speciation in Asia Minor and the Balkans as revealed from the phylogeny of spined loaches (Osteichthyes; Cobitidae). Molecular Phylogenetics and Evolution 39: 552–561. doi: 10.1016/j.ympev.2005.12.007 16439160 10.1016/j.ympev.2005.12.007

[B6] Chiari M Djeric N Garfagnoli F Hrvatović Krstić M Levi N Malasoma A Marroni M Menna F Nirta G Pandolfi L Principi G Saccani E Stojadinovi Trivić B (2011) The geology of the Zlatibor-Maljen area (Western Serbia): a geotraverse across the ophiolites of the Dinaric-Hellenic collisional belt. Ofioliti 36(2): 139–166.

[B7] Conti L Schmidt-Kloiber A Grenouillet G Graf W (2013) A trait-based approach to assess the vulnerability of European aquatic insects to climate change. Hydrobiologia 721(1): 297–315. doi: 10.1007/s10750-013-1690-7

[B8] Deltshev C (2008) Faunisitc diversity and zoogeography of cave-dwelling spiders on the Balkan Peninsula. Advances in Arachnology and Developmental Biology 12: 327–348.

[B9] Dudgeon D Arthington AH Gessner MO Kawataba ZI Knowler DJ Lévêque C Naiman RJ Prieur-Richard AH Soto D Stiassny MLJ Sullivan CA (2006) Freshwater biodiversity: importance, threats, status and conservation challenges. Biological Reviews 81: 163–182. doi: 10.1017/S1464793105006950 16336747 10.1017/S1464793105006950

[B10] Eastwood WJ (2004) East Mediterranean vegetation and climate change. In: Griffiths HI Kryštufek B Reed J (Eds) Balkan Biodiversity. Kluwer Academic Publishers, Dordrecht, 25–48. doi: 10.1007/978-1-4020-2854-0_3

[B11] Foster GN (1991) Conserving insects of aquatic and wetland habitats, with special reference to beetles. In: Collins NM Thomas JA (Eds) The conservation of insects and their habitats. Academic Press, London, 237–262. doi: 10.1002/rrr.3450070310

[B12] Freyhof J (2012) Threatened freshwater fishes and molluscs of the Balkan, potential impact of hydropower projects. Unpublished report, ECA Watch Austria&EuroNatur, 81 pp.

[B13] Geismar J Haase P Nowak C Sauer J Pauls SU (2015) Local population genetic structure of the montane caddisfly *Drusus discolor* is driven by overland dispersal and spatial scaling. Freshwater Biology 60: 209–221. doi: 10.1111/fwb.12489

[B14] Graf W Murphy J Dahl J Zamora-Muñoz C López-Rodríguez MJ (2008) Volume 1 - Trichoptera. In: Schmidt-Kloiber A, Hering D (Eds), Distribution and Ecological Preferences of European Freshwater Organisms. Pensoft Publishers, Sofia, Moscow, 388 pp.

[B15] Graf W Waringer Pauls J (2009) A new feeding group within larval Drusinae (Trichoptera: Limnephilidae): the *Drusus alpinus* Group sensu Schmid, 1956, including larval descriptions of *Drusus franzi* Schmid, 1956, and *Drusus alpinus* (Meyer-Dür, 1875). Zootaxa 2031: 53–62. PMC4826607 27069350

[B16] Griffiths HI Frogley MR (2004) Fossil ostracods, faunistics and the evolution of regional biodiversity. In: Griffiths HI Kryštufek B Reed J (Eds) Balkan Biodiversity. Kluwer Academic Publishers, Dordrecht, 261–272. doi: 10.1007/978-1-4020-2854-0

[B17] Griffiths HI Krystufek B Reed JM (2004) Balkan Biodiversity. Kluwer Academic Publishers, Dordrecht, 355 pp. doi: 10.1007/978-1-4020-2854-0

[B18] Guéorguiev B (2007) Biogeography of the endemic Carabidae (Coleoptera) in the Central and Eastern Balkan Peninsula. In: Fet V Popov E (Eds) Biogeography and Ecology of Bulgaria, Springer, Dordrecht, 297–356. doi: 10.1007/978-1-4020-5781-6_9

[B19] Hering D Schmidt-Kloiber A Murphy J Lucke S Zamora-Muñoz C Lopez-Rodriguez MJ Huber T Graf W (2009) Potential impact of climate change on aquatic insects: A sensitivity analysis for European caddisflies (Trichoptera) based on distribution patterns and ecological preferences. Aquatic Sciences 71: 3–14. doi: 10.1007/s00027-009-9159-5

[B20] Ibrahimi H Kučinić M Vitecek S Waringer J Graf W Previšić A Bálint M Keresztes L Pauls S (pers. comm.) New records for the Kosovo caddisfly fauna with the description of a new species, *Drusus dardanicus* sp. nov. (Trichoptera: Limnephilidae). Submitted for publication at Zootaxa. 10.11646/zootaxa.4032.5.5 PMC4975516 26624385

[B21] Illies J (1978) Limnofauna Europaea. A Checklist of the Animals Inhabiting European Inland Waters, with an Account of their Distribution and Ecology. 2nd Edition, Gustav Fischer Verlag, Stuttgart, 552 pp.

[B22] Jaskuła R (2011) How unique is the tiger beetle fauna (Coleoptera, Cicindelidae) of the Balkan Peninsula? ZooKeys 100: 487–502. doi: 10.3897/zookeys.100.1542 21738429 10.3897/zookeys.100.1542 PMC3131033

[B23] Karaman I Hammouti N Pavićević D Kiefer A Horvatović M Seitz A (2011) The genus *Troglophilus* Krauss, 1879 (Orthoptera, Rhaphidophoridae) in the west Balkans. Zoological Journal of the Linnean Society 163: 1035–1063. doi: 10.1111/j.1096-3642.2011.00738.x

[B24] Kenyeres Z Rácz IA Varga Z (2009) Endemism hot spots, core areas and disjunctions in European Orthoptera. Acta Zoologica Cracoviensia 52B(1-2): 189–211. doi: 10.3409/azc.52b_1-2.189-211

[B25] Klobučar GIV Podnar M Jelić M Franjević D Faller M Štambuk A Gottstein S Simić V Maguire I (2013) Role of the Dinaric karst (Western Balkans) in shaping the phylogeographic structure of the threatened crayfish *Austropotamobius torrentium*. Freshwater Biology 58: 1089–1105. doi: 10.1111/fwb.12110

[B26] Kryštufek B (2004) A quantitative assessment of Balkan mammal diversity. In: Griffiths HI Kryštufek B Reed J (Eds) Balkan Biodiversity. Kluwer Academic Publishers, Dordrecht, 79–108. doi: 10.1007/978-1-4020-2854-0_6

[B27] Kučinić M Previšić A Graf W Šeric Jelaska L Stanić-Koštroman S Waringer J (2011a) Larval description, genetic and ecological features of *Drusus radovanovici radovanovici* Marinković-Gospodnetić, 1971 (Trichoptera: Limnephilidae) with some phylogenetic and taxonomic data on the *bosnicus* group in the Balkan Peninsula. Deutsche Entomologische Zeitschrift 58: 136–153. doi: 10.1002/mmnd.201100010

[B28] Kučinić M Previšić A Stanić-Koštroman S Graf W Franjević M Posilović H Waringer J (2011b) Morphological and ecological features of *Drusus* larvae from the *bosnicus* group on the Balkan Peninsula with description of the larva of *Drusus klapaleki* Marinković-Gospodnetić, 1976. Zoosymposia 5: 244–254.

[B29] Kumanski K (1973) Die Unterfamilie Drusinae (Trichoptera) in Bulgarien. Tijdschrift voor Entomologie 116: 107–121.

[B30] Malicky H (2004) Atlas of European Trichoptera, Second edition Springer, 359 pp.

[B31] Malicky H (2005) Ein kommentiertes Verzeichnis der Köcherfliegen (Trichoptera) Europas und des Mediterrangebietes. Linzer biologische Beiträge 37: 533–596.

[B32] Marinković-Gospodnetić M (1976) The differentiation of *Drusus* species of the group *bosnicus*. In: Malicky H (Ed.) Proceedings of the First International Symposium on Trichoptera, Dr. W. Junk Publishers, The Hague, 77–85. doi: 10.1007/978-94-010-1579-0_13

[B33] Médail F Diadema K (2009) Glacial refugia influence platn diversity patterns in the Mediterranean Basin. Journal of Biogeography 36: 1333–1345. doi: 10.1111/j.1365-2699.2008.02051.x

[B34] Mereďa P Jr. Hoálová I Kučera J Zozomová-Lihová J Letz DR Slovák M (2011) Genetic and morphological variation in *Viola suavis* s.l. (Violaceae) in the Western Balkan Peninsula: two endemic subspecies revealed. Systematics and Biodiversity 9(3): 211–231. doi: 10.1080/14772000.2011.603903

[B35] Müller-Peddinghaus EH (2011) Flight-morphology of Central European caddisflies (Insecta: Trichoptera) in relation to their ecological preferences. Dr. rer. nat., University of Duisburg-Essen, Essen.

[B36] Neubauer F (2002) Evolution of late Neopoterozoic to early Paleozoic tectonic elements in Central and Southeast European Alpine mountain belts: review and synthesis. Tectonophysics 352: 87–103. doi: 10.1016/S0040-1951(02)00190-7

[B37] Nielsen A (1957) A comparative study of the genital segments and their appendages in male trichoptera. Biologiske Skrifter udgivet af Det Kongelige Dankse Vedenskabernes Sleskab 8: 1–159.

[B38] Oláh J (2010) New species and new records of Palearctic Trichoptera in the material of the Hungarian Natural History Museum. Annales Historico-Naturales Musei Nationalis Hungarici 102: 65–117.

[B39] Oláh J (2011) New species and records of Balkan Trichoptera. Folia Historico Naturalia Musei Matrensis 35: 111–121.

[B40] Oláh J Kovács T (2013) New species and new records of Balkan Trichoptera II. Folia Historico Naturalia Musei Matrensis 37: 109–121.

[B41] Pauls SU Lumbsch HT Haase P (2006) : Phylogeography of the montane caddisfly *Drusus discolor*: Evidence for multiple refugia and periglacial survival. Molecular Ecology 15: 2153–2169. doi: 10.1111/j.1365-294X.2006.02916.x 16780432 10.1111/j.1365-294X.2006.02916.x

[B42] Pauls SU Graf W Haase P Lumbsch HT Waringer J (2008) Grazers, shredders and filtering carnivores - The evolution of feeding ecology in Drusinae (Trichoptera: Limnephilidae): Insights from a molecular phylogeny. Molecular Phylogenetics and Evolution 46: 776–791. doi: 10.1016/j.ympev.2007.11.003 18171625 10.1016/j.ympev.2007.11.003 PMC4789499

[B43] Pauls SU Theißinger K Ujvarosi L Bálint M Haase P (2009) Population structure in two closely related, partially sympatric caddisflies in Eastern Europe: historic introgression, limited dispersal and cryptic diversity. Journal of the North American Benthological Society 28: 517–536. doi: 10.1899/08-100.1

[B44] Pešić V Glöer P (2013) A new freshwater snail genus (Hydrobiidae, Gastropoda) from Montenegro, with a discussion on gastropod diversity and endemism in Skadar Lake. ZooKeys 281: 69–90. doi: 10.3897/zookeys.281.4409 23794834 10.3897/zookeys.281.4409 PMC3677384

[B45] Petkovski T Scharf BW Keyser D (2009) Freshwater Ostracode (Crustacea) collected from caves and the interstitial habitat in Herzegovina, NW Balkan, with the description of two new species. Bulletin de la Société des naturalistes luxembourgeois 110: 173–182.

[B46] Petrova A Vladimirov V (2010) Balkan endemics in the Bulgarian flora. Phytologia Balcanica 16(2): 293–311.

[B47] Polhemus DA (1993) Conservation of Aquatic Insects: Worldwide crisis or worldwide threats? American Zoologist 33(6): 588–598.

[B48] Previšić A Walton C Kučinić M Mitrikeski PT Kerovec M (2009) Pleistocene divergence of Dinaric *Drusus* endemics (Trichoptera, Limnephilidae) in multiple microrefugia within the Balkan Peninsula. Molecular Ecology 18: 634–647. doi: 10.1111/j.1365-294X.2008.04046.x 19175506 10.1111/j.1365-294X.2008.04046.x

[B49] Previšić A Graf W Vitecek S Kučinić M Bálint M Keresztes L Pauls SU Waringer J (2014a) Cryptic diversity of caddisflies in the Balkans: the curious case of *Ecclisopteryx* species (Trichoptera: Limnephilidae). Arthropod Systematics and Phylogeny 72(3): 309–329. 25810791 PMC4370265

[B50] Previšić A Schnitzler J Kučinić M Graf W Ibrahimi H Kerovec M Pauls SU (2014b) Microscale vicariance and diversification of western Balkan caddisflies linked to karstification. Freshwater Science 33: 250–262. doi: 10.1086/674430 10.1086/674430 PMC4813752 27042385

[B51] Redžić S (2011) Phytogeographic and syntaxonomic diversity of high mountain vegetation in Dinaric Alps (Western Balkan, SE Europe). Journal of Mountain Science 8: 767–786. doi: 10.1007/s11629-011-2047-1

[B52] Reed JM Kryštufek B Eastwood WJ (2004) The physical geography of the Balkans and nomenclature of place names. In: Griffiths HI Kryštufek B Reed J (Eds) Balkan Biodiversity. Kluwer Academic Publishers, Dordrecht, 9–23. doi: 10.1007/978-1-4020-2854-0_2

[B53] Schmid F (1956) La sous-famille des Drusinae (Trichoptera, Limnophilidae). Mémoires de l’Institut Royal des Sciences Naturelles de Belgique, 2. serie, 55: 1–92.

[B54] Schmidt-Kloiber A Hering D (Eds) (2012) www.freshwaterecology.info - the taxa and autecology database for freshwater organisms, version 5.0 [accessed on 23.04.2015]

[B55] Schwarz U (2012) Balkan Rivers - The Blue Heart of Europe. Hydromorphological Status and Dam Projects. Report. Vienna, Austria, 151 pp.

[B56] Snodgrass RE (1935) Principles of Insect Morphology. Cornell University Press, Ithaca, New York, 667 pp.

[B57] Stevanović V Vukojičić S Šinžar-Sekulić J Lazarević M Tomović G Tan K (2009) Distribution and diversity of Arctic-Alpine species in the Balkans. Plant Systematics and Evolution 283: 219–235. doi: 10.1007/s00606-009-0230-4

[B58] Thomson RE Holzenthal RW (2010) New Neotropical species of the genus *Austrotinodes* Schmid (Trichoptera: Ecnomidae). Zootaxa 2437: 38–50.

[B59] Tierno de Figueroa JM López-Ródriguez MJ Lorenz A Graf W Schmidt-Kloiber A Hering D (2010) Vulnerable taxa of European Plecoptera (Insecta) in the context of climate change. Biodiversity and Conservation 19(5): 1269–1277. doi: 10.1007/s10531-009-9753-9

[B60] Tzedakis PC (2004) The Balkans as prime glacial refugial territory of European temperate trees. In: Griffiths HI Kryštufek B Reed J (Eds) Balkan Biodiversity. Kluwer Academic Publishers, Dordrecht, 49–68. doi: 10.1007/978-1-4020-2854-0_4

[B61] Tzedakis PC (2009) Museums and cradles of Mediterranean biodiversity. Journal of Biogeography 36: 1033–1034. doi: 10.1111/j.1365-2699.2009.02123.x

[B62] Ursenbacher S Schweiger S Tomović L Crnobrnja-Isailović J Fumigalli L Mayer W (2008) Molecular phylogeography of the nose-horned viper (*Vipera ammodytes*, Linnaeus (1758)): Evidence for high genetic diversity and multiple refugia in the Balkan Peninsula. Molecular Phlyogenetics and Evolution 46: 1116–1128. doi: 10.1016/j.ympev.2007.11.002 10.1016/j.ympev.2007.11.002 18267369

[B63] Vitecek S Graf W Previšić A Kučinić M Oláh J Bálint M Keresztes L Pauls S Waringer J (in press) A hairy case: The evolution of filtering carnivorous Drusinae (Limnephilidae, Trichoptera). Molecular Phylogenetics and Evolution. 10.1016/j.ympev.2015.07.019 PMC4783861 26265260

[B64] Vitecek S Previšić A Kučinić M Bálint M Keresztes L Waringer J Pauls S Malicky H Graf W (2015) Description of a new species of *Wormaldia* from Sardinia and a new *Drusus* species from the Western Balkans (Trichoptera, Philopotamidae, Limnephilidae). ZooKeys 496: 85–103. doi: 10.3897/zookeys.496.9169 25931956 10.3897/zookeys.496.9169 PMC4410158

[B65] Waringer J Graf W (2011) Atlas of Central European Trichoptera Larvae. Erik Mauch Verlag, Dinkelscherben, 468 pp.

[B66] Weiss M Macher JN Seefeldt MA Leese F (2014) Molecular evidence for further overlooked species within the *Gammarus fossarum* complex (Crustacea: Amphipoda). Hydrobiologia 721: 165–184. doi: 10.1007/s10750-013-1658-7

[B67] Wiggins GB (1998) Larvae of the North American Caddisfly Genera (Trichoptera), University of Toronto Press, Toronto, 457 pp.

[B68] Wilke T Schultheiß R Albrecht C Bornmann N Trajanovski S Kevrekidis T (2010) Native *Dreissena* freshwater mussels in the Balkans: in and out of ancient lakes. Biogeosciences 7: 3051–3065. doi: 10.5194/bg-7-3051-2010

[B69] Zakšek V Sket B Gottstein S Franjević D Trontelj P (2009) The limits of cryptic diversity in groundwater: phylogeography of the cave shrimp *Troglocaris anophtalmus* (Crustacea: Decapoda: Atyidae). Freshwater Biology 18: 931–946. 10.1111/j.1365-294X.2008.04061.x 19207253

[B70] Zarfl C Lumsdon AE Berlekamp J Tydecks L Tockner K (2014) A global boom in hydropower dam construction. Aquatic Sciences 77(1): 161–170. doi: 10.1007/s00027-014-0377-0

[B71] Zogaris S Economoi AN Dimopoulos P (2009) Ecoregions in the Southern Balkans: Should their boundaries be revised? Environmental Management 43(4): 682–697. doi: 10.1007/s00267-008-9243-y 19145399 10.1007/s00267-008-9243-y

